# Correction: Zhang, N.; Lin, C. The Image Definition Assessment of Optoelectronic Tracking Equipment Based on the BRISQUE Algorithm with Gaussian Weights. *Sensors* 2023, *23*, 1621

**DOI:** 10.3390/s23146323

**Published:** 2023-07-12

**Authors:** Ning Zhang, Cui Lin

**Affiliations:** 1Changchun Institute of Optics, Fine Mechanics and Physics, Chinese Academy of Sciences, Changchun 130033, China; ning0025@163.com; 2University of Chinese Academy of Sciences, Beijing 100049, China

## References

There was an error in the original publication [1].

There are some problems with the order of references cited in the original publication. Corrections need to be made.

The specific order of reference changes is as follows:

The original [29] becomes the current [31]:31.Han, L.; Lv, H.; Zhao, Y.; Liu, H.; Bi, G.; Yin, Z.; Fang, Y. Conv-Former: A Novel Network Combining Convolution and Self-Attetion for Image Quality Assessment. *Sensors*
**2023**, *23*, 427.

The original [30] becomes the current [29]:29.Mittal, A.; Soundararajan, R.; Bovik, A.C. Making a “Completely Blind” Image Quality Analyzer. *IEEE Signal Process. Lett.*
**2013**, *20*, 209–212.

The original [31] becomes the current [30]:30.Li, C.; Bovik, A.C.;Wu, X. Blind Image Quality Assessment Using a General Regression Neural Network. *IEEE Trans. Neural Netw.*
**2011**, *22*, 793–799.

The original [32] becomes the current [34]:34.Varga, D. No-Reference Video Quality Assessment Using the Temporal Statistics of Global and Local Image Features. *Sensors*
**2022**, *22*, 9696.

The original [33] becomes the current [32]:32.Ruderman, D.L. The Statistics of Natural Images. *Netw. Comput. Neural Syst.*
**1994**, *5*, 517–548.

The original [34] becomes the current [33]:33.Simoncelli, E.P.; Freeman, W.T.; Adelson, E.H.; Heeger, D.J. Shiftable Multiscale Transforms. *IEEE Trans. Inf. Theory*
**1992**, *38*, 587–607.

A correction has been made to “Order of references”

Ruderman et al. found that the luminance of natural image normalization tends to follow a normal (Gaussian) distribution [32]. They posit that the distortion of an image changes the statistical characteristics of the normalization coefficient. By measuring the changes in the statistical characteristics, the distortion type can be predicted and the image visual quality can be evaluated [33]. Based on this theory, Mr. Mittal put forward the BRISQUE algorithm [28], which is based on the image spatial statistical characteristics. Ronin Institute et al. apply a broad spectrum of statistics of local and global features to characterize the variety of possible video distortions [34].

The authors state that the scientific conclusions are unaffected. This correction was approved by the Academic Editor. The original publication has also been updated.
